# Prediction of the aggregation rate of nanoparticles in porous media in the diffusion-controlled regime

**DOI:** 10.1038/s41598-023-50643-x

**Published:** 2024-01-22

**Authors:** Vi T. Nguyen, Ngoc H. Pham, Dimitrios V. Papavassiliou

**Affiliations:** School of Sustainable Chemical, Biological and Materials Engineering, The University of Oklahoma, Norman, OK 73019 USA

**Keywords:** Chemical engineering, Nanoparticles

## Abstract

The fate and aggregation of nanoparticles (NPs) in the subsurface are important due to potentially harmful impacts on the environment and human health. This study aims to investigate the effects of flow velocity, particle size, and particle concentration on the aggregation rate of NPs in a diffusion-limited regime and build an equation to predict the aggregation rate when NPs move in the pore space between randomly packed spheres (including mono-disperse, bi-disperse, and tri-disperse spheres). The flow of 0.2 M potassium chloride (KCl) through the random sphere packings was simulated by the lattice Boltzmann method (LBM). The movement and aggregation of cerium oxide (CeO_2_) particles were then examined by using a Lagrangian particle tracking method based on a force balance approach. This method relied on Newton's second law of motion and took the interaction forces among particles into account. The aggregation rate of NPs was found to depend linearly on time, and the slope of the line was a power function of the particle concentration, the Reynolds (*Re*) and Schmidt (*Sc*) numbers. The exponent for the *Sc* number was triple that of the *Re* number, which was evidence that the random movement of NPs has a much stronger effect on the rate of diffusion-controlled aggregation than the convection.

## Introduction

The transport of nanoparticles (NPs) in porous media has applications across various fields due to the NP distinctive properties at the nanoscale^1–6^. NPs can be injected into hydrocarbon reservoirs to improve the oil flow in enhanced oil recovery^7–9^ and can be utilized to treat different pollutants in underground water remediation^5,10–13^. In biomedical applications, nanoparticle-based drugs can be delivered through porous tissue structures to reach specific targets in the body^8,14,15^. The effectiveness of such applications is advanced by understanding the mobility of NPs in porous media. Aggregation of NPs can significantly impact mobility because the creation of large aggregates often obstructs the pore space^16–18^.

However, aggregation is not well understood mainly due to challenges associated with performing experiments and taking measurements inside porous media^18,19^. Consequently, there have been many computational models built to simulate the aggregation process in porous media^20–28^. The most challenging problem of such models arises from the multiscale nature of the aggregation process. It is a combination of the movement of NPs and interactions among NPs, which are simulated at large (10^−5^–10^−4^ s) and small (10^−9^–10^−8^ s) timesteps, respectively^25,29^. If the model relies on the physics of particle interactions, the use of very small timesteps is required to ensure the accurate simulation of the aggregation process; however, this results in a remarkable increase in the simulation time and it is impractical to simulate the process for a sufficiently long period to observe significant aggregation^29^. Therefore, most models have used large timesteps to study the movement of particles in porous media and utilized probabilities for particle attachment and collision to predict the aggregation of NPs in porous media^1,17,25,27^. Many researchers have utilized the Smoluchowski model^20–25^ and Monte Carlo methods^3,26,27,30^ to predict the aggregation kinetics of nanoparticles in porous media.

The Smoluchowski model accounts for the aggregation of nanoparticles as a second-order process. The change in the concentration of a cluster containing k primary particles with time $t$ is described as follows:^21,31^

1 $$\frac{{dn}_{k}}{dt}=\frac{1}{2}\alpha \sum_{i+j=k}\beta \left(i,j\right){n}_{i}{n}_{j}-\alpha {n}_{k}\sum_{i=1}^{c}\beta (i,k){n}_{i}$$

The concentrations of particles i, j, and k are shown as ${n}_{i}$, ${n}_{j}$, ${n}_{k}$, respectively, while the maximum number of primary particles in an aggregate is described as c^21,31^. The collision efficiency $\alpha$ represents the probability that two particles attach to each other after collision. The collision efficiency is equal to 1 if the two particles always aggregate upon a collision. The value of the collision efficiency $\alpha$ is dependent on the physicochemical interactions among particles; however, it is difficult to find the relationship between the collision efficiency and the interactions among particles. Thus, most researchers have varied the value of $\alpha$ to make their simulation results match experimental data. As regards to the collision mechanism, it is represented by the collision frequency function $\beta$. In this model, the collision is caused by three mechanisms: Brownian motion, fluid shear stress, and size differences among particles. Thus, the collision frequency function $\beta$ can be determined based on the molecular diffusivity of particles, the fluid velocity, and the size of particles^21,31^. The Smoluchowski model is often combined with the advection–dispersion equation^24^ or the Lagrangian particle tracking (LPT) method^17,25^ to estimate the aggregation of NPs while they move in porous media. In LPT method, particle movement is a result of convection and molecular diffusion caused by the fluid velocity and the Brownian motion of the NPs, respectively^32,33^, while particle interactions are ignored.

Monte Carlo is widely recognized as a probability-based method for estimating the movement and transport of particles^27,30^. Hul et al. devised a Monte Carlo model with off-lattice coarse-graining to explore the aggregation and transportation of nanoparticles in porous media^27^. In their study, the displacement of individual particles at each time step was determined by a random angle and distance within 5 nm, and particles were restricted from moving backward in the flow direction. At the initiation of each simulation, particle–particle and particle-collector attachment efficiencies were pre-determined. When there was a collision between two particles or between a particle and a collector, a random number between 0 and 1 was generated that followed a uniform distribution. If the random number was less than the pre-defined attachment efficiency, the particle was considered to attach to the other particle or the collector.

To obtain estimates of the aggregation or collision probabilities that are needed to implement such stochastic models, it is important to perform experiments to ensure that the simulation data agree with the experimental ones. Otherwise, such results are not reliable. However, performing many experiments related to aggregation of NPs in porous media is an arduous and challenging task. Thus, building a model relying on the physics of particle interactions is important to achieve reliable results and to reduce experiment time and resources.

The size of aggregates and the aggregation kinetics have been reported to depend on many factors including the electrolyte concentration in the NP suspension^18,31,34–37^, the size of primary particles^38,39^, the fluid velocity^17,25,40^, the particle concentration^17,24,41,42^, and so on. Li et al.^36^ conducted experiments with CeO_2_ NPs in aqueous suspensions of different KCl concentrations and found that the aggregation rate was proportional to the electrolyte concentration; however, when the electrolyte concentration was higher than the critical coagulation concentration (CCC), the aggregation fell in the diffusion-limited regime and the aggregation rates did not change with the electrolyte concentration. The reason was that when the salt concentration increased before reaching the CCC, the more ions moved freely in the solution. These ions could shield the electric field between charged particles, thus reducing the strength of the electrostatic force^35,43,44^. Consequently, the energy barrier for two particles aggregating with each other decreased when the electrolyte concentration increased^36^. Aggregation occurring when the salt concentration was below the CCC is called reaction-limited aggregation. Once the concentration was equal to or higher than the CCC, there was no energy barrier and the attachment efficiency reached unity. In this case, the aggregation rate was controlled by the diffusion, thus aggregation was in the diffusion-limited regime.

Pham and Papavassiliou^25^ utilized the lattice Boltzmann method in conjunction with a Lagrangian particle tracking method to simulate the movement of nanoparticles through randomly packed spheres. The aggregation of nanoparticles was predicted based on the collision and attachment probabilities, which were found by matching with experimental results. The NP aggregation rate was found to be a function of time when time was normalized using the time needed to drain the porous medium (i.e., the pore volume (PV) unit)^25,29^. The aggregation rate at high fluid velocity was found to be smaller than that at low velocity because the residence time in porous media for particles traveling at high velocity was small and they did not have many chances to collide with other particles^29^.

Regarding effects of particle size, at the same electrolyte concentration and particle concentration, the high molecular diffusion of small particles promoted collision efficiency and thus aggregation rate^38,39^. Raychoudhury et al.^24^ performed experiments investigating the effect of nanoparticle concentration on the aggregation of carboxymethyl cellulose-modified nanoparticles of zero-valent iron (NZVI) in 0.1 mM NaHCO_3_ in a packed column. At very low particle concentrations, the particle size did not change with time, while at higher concentrations, the particle size increased significantly with time due to aggregation among particles^24^. When the particle concentration increased, there was a high chance for particles to be in interacting zones with others and form aggregates. In brief, it was found that the fluid velocity, particle size, and particle concentration had remarkable effects on the aggregation kinetics at the same electrolyte concentration. Nonetheless, most prior work did not show how to predict and control the aggregation rate using these parameters leaving a gap in aggregation understanding.

Despite the complexity of geometries and fluid flow in porous media, the local fluid velocities, fluid stresses^45–48^, and particle velocities^49,50^ in porous media could be predicted. For example, Voronov et al.^47^ found a universal three-parameter Gamma probability density function that can be used to predict the wall stress distribution in different scaffolds with porosity higher than 80%. Given the above observations, one can wonder whether the aggregation rate of NPs in porous media can be estimated. Therefore, it is important to find an equation showing the dependence of aggregation kinetics or aggregate size on time, particle concentration, Reynolds (*Re*), and Schmidt (*Sc*) numbers. The nanoparticles investigated in our work are cerium oxide (CeO_2_) that has been one of the most employed NPs in manufacturing gas sensors, combustion catalysts, solid oxide fuel cells, and so on^51^. Given their extensive applications, the release of CeO_2_ NPs is common, which could potentially lead to toxic effects on the environment such as inhibiting soybean growth^52^, causing DNA lesions^53^, and toxicity in human lung cells^54^. Therefore, understanding the aggregation and mobility of NPs like CeO_2_ is critical.

In brief, the contribution of our work is to model the aggregation rate of NPs in high electrolyte concentration solutions which is based on the physics of particle interactions. The primary particles in this work are spherical, and the aggregation is considered as a one-way coupling process. The model predicts aggregation rate as a function of time, NP concentration, *Re* and *Sc* numbers as they propagate in the pores between randomly packed spheres including mono-disperse, bi-disperse, and tri-disperse sphere packings. The lattice Boltzmann method (LBM) is applied to calculate the velocity field of an aqueous solution in porous media at the steady state; and the Lagrangian particle tracking with force balance (LPT/FB) method is then applied to keep track of the positions and velocities of particles at each time step^29^. The positions of particles at each step are used to compute the hydrodynamic radius of aggregates. The LPT/FB method utilizes a force balance approach, relying on Newton’s second law of motion and taking the interaction forces among particles into consideration. Thus, the aggregation process of NPs in sphere packings is accurately simulated without using probabilistic methods. CeO_2_ NPs in 0.2 M KCl aqueous solution were simulated because at this electrolyte concentration, the aggregation of CeO_2_ NPs was found to be in the diffusion-limited regime, as proven by both experimental and simulation results^29,36^.

## Methods

### Lattice Boltzmann method

The lattice Boltzmann method^55–57^ is employed herein to simulate the flow of fluid in porous media and obtain the velocity field at the steady state. In this method, the simulation domain is divided into a cubic lattice. Each node in the lattice is represented by a binary value which is equal to “True” for solid nodes and “False” for fluid nodes. The basis of this method is to apply the discretized Boltzmann equation in conjunction with mass and momentum conservation equations to compute the fluid velocities on the fluid nodes. The Boltzmann equation is a model that calculates changes in the particle distribution function when fluid particles move among the fluid nodes via streaming, collision, and forcing steps. The flow is induced by specifying a pressure drop (i.e., a forcing factor) along the flow direction. The no-slip and periodic boundary conditions are applied for the wall-fluid interfaces and the six faces of the three-dimensional computational domain, respectively. An in-house code^55,58^ based on this method was written and has undergone careful validation for use in porous media, comparing its results to the Blake-Kozeny equation^59,60^, simulation data^59,61,62^, and experimental outcomes from other groups^45,59,63^.

### The Lagrangian particle tracking method with force balance

While most models simulate the aggregation of particles by applying the Smoluchowski model^21,31^ together with either a conventional LPT method^25,32^ or an advection–dispersion equation^24,64^, our LPT/FB method^29^ takes the interactions among NPs into account by using a force balance approach. Newton’s second law of motion is applied for each particle with mass ${m}_{p}$ and velocity $\overrightarrow{{V}_{p}}$ as follows:^29,65^

2 $${m}_{p}\frac{d\overrightarrow{{V}_{p}}}{dt}=\overrightarrow{{F}_{g}}+\overrightarrow{{F}_{b}}+\overrightarrow{{F}_{d}}+\overrightarrow{{F}_{r}}+\overrightarrow{{F}_{e}}+{\overrightarrow{F }}_{vdW}.$$

There are six forces exerted on each particle, including the gravity force ($\overrightarrow{{F}_{g}}$)^66^, buoyancy force ($\overrightarrow{{F}_{b}}$)^66^, drag force ($\overrightarrow{{F}_{d}}$)^31,66,67^, random force ($\overrightarrow{{F}_{r}}$)^68^, electrostatic force ($\overrightarrow{{F}_{e}}$)^12,39,69^, and van der Waals force (${\overrightarrow{F }}_{vdW}$)^12,39,69^. The magnitudes of these forces are calculated as seen below:

3 $$\left|\overrightarrow{{F}_{g}}\right|=\frac{\pi }{6}{\rho }_{p}g{D}^{3}$$

4 $$\left|\overrightarrow{{F}_{b}}\right|=\frac{\pi }{6}{\rho }_{f}g{D}^{3}$$

5 $$\left|\overrightarrow{{F}_{d}}\right|=3\pi \mu D(U-{V}_{p})$$

6 $$\left|\overrightarrow{{F}_{r}}\right|=\sqrt{\frac{6\pi {k}_{B}TD\mu }{dt}}\upxi$$

7 $$\left|\overrightarrow{{F}_{e}}\right|=2\pi \varepsilon {\varepsilon }_{0}{\psi }^{2}R\left(\frac{k{e}^{-kh}}{1+{e}^{-kh}}\right)$$

8 $$k= \sqrt{\frac{2{N}_{A}I{e}_{o}^{2}}{\varepsilon {\varepsilon }_{0}{k}_{B}T}}$$

9 $$\left|{\overrightarrow{F }}_{vdW}\right|=\frac{32A}{3}\left[\frac{{R}^{6}}{{\left(2R+h\right)}^{3}{\left(4Rh+{h}^{2}\right)}^{2}}\right].$$

where ${\rho }_{p}$ and ${\rho }_{f}$ are the densities of NPs and fluid, respectively; $g$ is the gravity constant; $D$ and $R$ are the diameter and radius of NPs, respectively; $U$ is the fluid velocity at the position of the particle and is calculated by the LBM; ${V}_{p}$ is the velocity of the particle; $\mu$ is the fluid dynamic viscosity; ${k}_{B}$ is the Boltzmann constant (1.38 × 10^−23^ J/K); *T* is the absolute temperature (298 K); $\upxi$ is a random variable that follows Gaussian distribution with a mean of zero and a variance of one; $dt$ is the time interval;${\varepsilon }_{0}$ represents the permittivity of vacuum (8.854 × 10^−12^ CV^−^^1^ m^−^^1^); $\psi$ is the surface potential of particles within the solution; $\upvarepsilon$ is the relative dielectric constant of the fluid; $k$ is the inverse Debye length; ${N}_{A}$ is Avogadro’s number (6.02 × 10^−23^ mol^−^^1^); $I$ is the ionic strength; and ${e}_{o}$ is the unit charge (1.602 × 10^−^^19^ C);$A$ is the Hamaker constant; and $h$ is the separation distance between two particles. To simulate the aggregation of CeO_2_ NPs in 0.2 M KCl solution^14^, ε = 78.5 (for water)^36^, $\psi$ = 45 mV, and $A$ = 5.57 × 10^−20^ J (for CeO_2_)^14,36,70^ are used in this study.

The gravity force acts in the downward direction, opposing the buoyancy force. The drag force, a resistive force exerted by a fluid on a particle, has the direction opposite to the motion of the particle. Van der Waals and electrostatic forces act along the line connecting interacting particles and have opposite directions. The van der Waals force brings two particles closer, whereas the electrostatic force operates to drive them apart. Equation (2) is applied for each particle, which could be a single particle (not belonging in any cluster) or an aggregate. A single particle that does not belong in an aggregate may interact with many neighboring particles; thus, the electrostatic force in Eq. (2) is the summation of all the electrostatic forces arising from these interactions. The same principle applied for the van der Walls force in Eq. (2), while the remaining forces are calculated for one primary particle based on Eqs. (3–6). If a particle is an aggregate, the gravity, buoyance, electrostatic, and van der Waals forces are computed by summing up all the forces exerted on each constituent particle in the aggregate. However, the drag force and random force are determined for the entire cluster based on Eqs. (5–6) with the use of the hydrodynamic radius of that cluster. The velocity in Eq. (2) represents the velocity of the cluster, which is equal to the velocity of the individual primary particles within that cluster. The rotation movement of particles and the interaction forces between particles and the solid phase of the porous medium are not considered in this work.

At the beginning of each simulation step, the initial velocities and positions of all particles are known. The initial positions at each step serve as the basis for identifying the neighbors of each particle within an interaction zone and computing the number of aggregate clusters and their sizes in the system. Two particles will be considered neighbors and will interact if the distance between their centers falls below the cut-off radius, which is triple the diameter of single particles. The reason for selecting this cut-off radius has been presented elsewhere^29^. When the separation distance between two particles is smaller than the primary minimum of the interaction force, the attraction is too large for them to be separated afterwards; hence, they are considered to be in the same aggregate and they are considered to have no interactions with other particles within the same cluster. The value of the primary minimum is selected based on the interactions forces and DLVO energy charts, which were presented in a previous work^29^; and it is equal to 0.5 nm to ensure that the particles are permanently attached to each other^29^. Most aggregates have fractal-like shapes; thus, the fractal dimension is utilized to describe how densely single particles are distributed in a cluster while the hydrodynamic radius is commonly used to represent the size of clusters^21,71^. A dense cluster has a higher fractal dimension than a loose one. The value of fractal dimension, *D*_*f*_, ranges from 1 (for a cluster of particles on a straight line) to 3 (for a compact spherical shape). It is computed based on the following equation:^72–74^

10 $$n={k}_{f}{\left(\frac{{R}_{g}}{R}\right)}^{{D}_{f}}$$

where *n* is the number of primary particles in a cluster, ${k}_{f}$ is the scale factor, $R$ is the radius of single particles, and ${R}_{g}$ is the radius of the gyration of a cluster. ${R}_{g}$ is equal to the root mean squared distance between particles in a cluster and the center of mass of the aggregate.

The hydrodynamic radius of an aggregate, ${R}_{h}$*,* is the radius of a solid sphere that exhibits similar diffusion characteristics as the aggregate and it is computed based on the fractal dimension and the radius of gyration of a cluster as given below:^72,75^

11 $${R}_{h}= \frac{2({D}_{f}-1)\sqrt{({D}_{f}+2)}}{{D}_{f}^{1.5}}{R}_{g}.$$

Next, all the forces applied on each particle or cluster are calculated by Eqs. (3–9). Unlike LBM (which is an on-lattice method) where fluid particles have pre-defined movement directions, NPs in the LPT/FB algorithm have the freedom to move in any direction. Thus, all forces acting on x, y, and z directions are computed and incorporated into Eq. (2) to find the particle velocities in these directions. To determine the drag force, the fluid velocities at particle positions are required. The fluid velocity field from LBM, which is at steady state and does not change with time, is used to find the fluid velocities at the positions of particles by a three-dimensional interpolation in space.

To apply Eq. (2) to calculate new velocities and new positions as the simulation time advances, the value for the time step, *dt,* is required. If a relatively large and constant *dt* is used for all simulation steps, the computation cost will be low and it is possible to simulate up to the time required to observe large aggregates. However, the aggregation rate cannot be computed correctly since at the large *dt*, particles can pass through each other and many aggregation events can be missed. On the contrary, if a very small *dt* (10^−9^–10^−8^ s) is applied for all the steps, more aggregation events will be captured, but the computational cost is too high to simulate the aggregation process. Therefore, in our study, dynamic timesteps are used and the values of *dt* at various steps are different. When there are at least two particles close to each other and interact, a very small *dt* is used to ensure that they cannot move more than one-tenth of the distance that separates them and one-fifth of the grid resolution of the porous medium. Consequently, no particles can pass through each other, or overlap with others, or move into the solid phase of the porous medium. However, this approach is practical for systems with low concentrations of NPs. Otherwise, interactions become excessively frequent, necessitating a small timestep for the majority of the simulated time.

At this stage, *dt* is known, and by applying the first order discretization for the velocity derivative in Eq. (2), the new velocities and positions of all particles are computed. The positions of particles are employed to see if they overlap with others or move in the solid matrix of the porous medium. The value of *dt* will be reduced by one-half if overlapping occurs. In case a particle is found in the solid phase, it is bounced back to its previous position. The new positions and velocities at this time step are the initial ones for the next time step and the same calculations are repeated. In this way, the velocities, positions of all primary particles, and the size of aggregates are tracked with time. This method has been validated against the aggregation experiments of CeO_2_ NPs suspended in KCl solutions with different concentrations (0.001 M, 0.02 M, 0.1 M, 0.15 M, and 0.2 M). More details about this method and the validation have been presented elsewhere^29^.

This model is based on the physical processes of particle interactions and involves more complex calculations than models based on probabilities. Thus, LPT/FB requires more computational time compared to the probability-based methods, which utilize a probability tool to estimate aggregation events at large timesteps. However, in stochastic models, to obtain the correct probabilities and reliable results, many experiments have to be performed. When variables such as particle size, fluid velocity, or particle concentration change, the collision and/or aggregation probabilities also change. Thus, conducting new experiments is essential to find the correct probabilities, which can be a challenging and time-consuming process. On the contrary, in our model, once the Hamaker constant and the surface potential (for van der Waals and electrostatic forces calculations) are known, conducting additional experiments when particle size, fluid velocity, or NP concentration change is unnecessary.

### Scope of work

This study investigated the effect of fluid velocity, particle size, and particle concentration on the rate of diffusion-limited aggregation of CeO_2_ in 0.2 M KCl through sphere packings, which are similar to sand packing. CeO_2_ NPs were examined because of their potentially harmful effects to the environment. In addition, the Hamaker constant and surface potential of CeO_2_ were known and the aggregation process of CeO_2_ in bulk was validated in our previous study^29^. The comparability of the results is ensured by keeping the porous medium and particle interactions the same, and varying parameters such as fluid velocity, particle size, and particle concentrations. The fluid velocities were chosen to study the effect of *Re* in the laminar flow regime, while the particle size was changed to study the impacts of *Sc*. The particle concentration was within the range of low concentrations. The porous media were mono-disperse, bi-disperse and tri-disperse to establish that the findings were valid for different porous media configurations. Finally, the effects of the force field were investigated.

**Figure 1:**
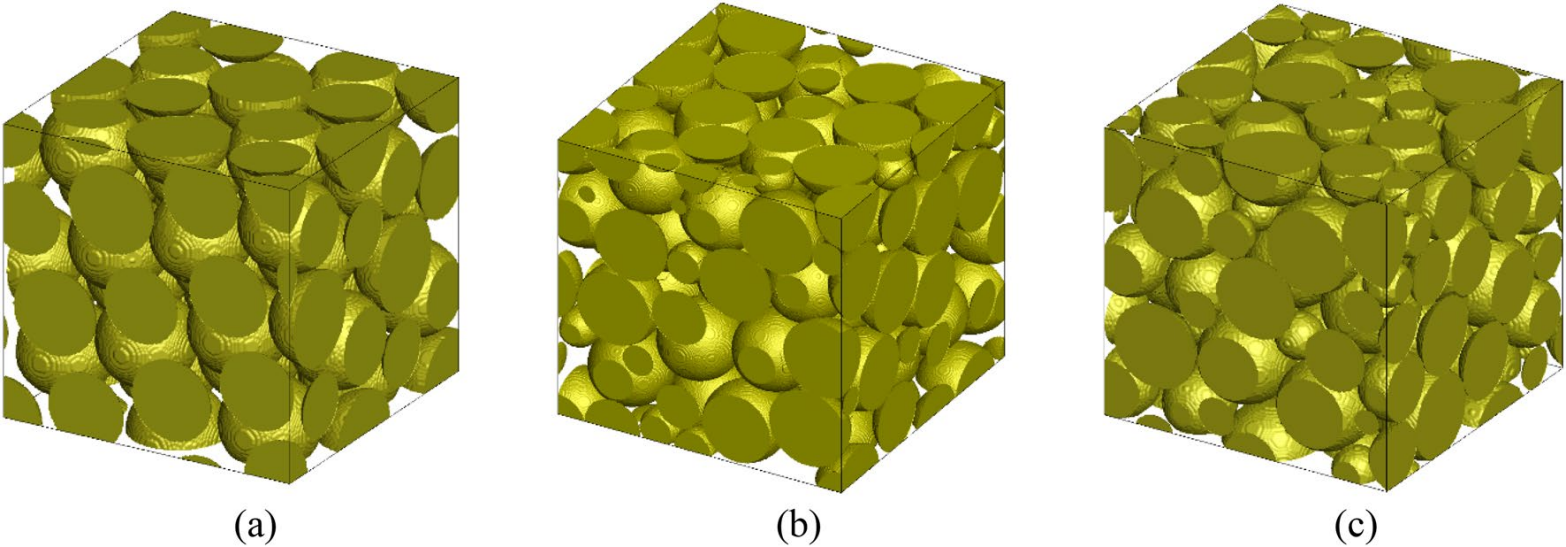
Geometries of random packings of (**a**) mono-disperse spheres, (**b**) bi-disperse spheres, and (**c**) tri-disperse spheres.

First, to explore how *Re* and *Sc* numbers affect the aggregation rate, twelve LBM runs were performed to simulate the flow of 0.2 M KCl solution through mono-disperse and bi-disperse sphere packings at six different pore velocities including 50, 100, 200, 500, 1000, and 2000 µm/s. Next, CeO_2_ NPs were uniformly released in the simulation domains; and the movement of NPs in porous media was then simulated by applying the LPT/FB method that accounted explicitly for interactions among particles. There were thirty LPT/FB simulations conducted for the aggregation of 10 mg/L CeO_2_ NPs in the mono-disperse sphere packing. For each pore velocity, five different LPT/FB runs were conducted with different values of particle radius (75, 85, 95, 105, and 110 nm), which corresponded to different numbers of NPs (21,428, 14,720, 10,544, 7809, and 6792) to ensure that the concentration of NPs was constant for all runs. Another thirty numerical experiments were repeated for the case of bi-disperse spheres. Results from these experiments were used to obtain an equation to predict the aggregation rate based on time, *Re*, and *Sc* numbers. There were six LPT/FB runs with different velocities, different particle sizes, and the same particle concentration carried out for the case of tri-disperse sphere packing to validate the model. They included experiments of 14,720 NPs sized 85 nm at fluid velocity 46 µm/s, 14,720 NPs sized 85 nm at fluid velocity 93 µm/s, 10,544 NPs sized 95 nm at pore velocity 93 µm/s, 7809 NPs sized 105 nm at pore velocity 93 µm/s, 10,544 NPs sized 95 nm at pore velocity 186 µm/s, and 7809 NPs sized 105 nm at pore velocity 1484 µm/s. The details of the three different types of randomly packed spheres investigated in this study are shown in Table 1 and Fig. 1. These sphere packings were created by utilizing the code of Baranau et al.^76,77^, which relied on the Lubachevsky–Stillinger generation algorithms^78,79^.

**Table 1:** Details of the simulation domains for mono-disperse sphere packing, bi-disperse sphere packing, and tri-disperse sphere packing (d_s_ is the diameter of the spheres used in the packing shown in Fig. 1).

Porous media	Grid points	Size (µm^3^)	Porosity (%)	Structure	Hydraulic diameter (µm)
Mono-disperse	401 × 401 × 401	418.9 × 418.9 × 418.9	37	40 spheres (d_s_ = 130.3 µm)	51.3
Bi-disperse	501 × 501 × 501	428.2 × 428.2 × 428.2	35	60 spheres (d_s_ = 115.2 µm) 30 spheres (d_s_ = 57.6 µm)	39.7
Tri-disperse	501 × 501 × 501	432.9 × 432.9 × 432.9	34	30 spheres (d_s_ = 130 µm) 30 spheres (d_s_ = 97.5 µm) 30 spheres (d_s_ = 65 µm)	39.1

To study the effects of NP concentration on the aggregation rate, NPs with seven different concentrations (3.6, 5.4, 7.4, 10.0, 14.0, 18.8, and 27.4 mg/L) were released in the mono-disperse sphere packing at three different pore velocities (100, 500, and 1000 µm/s) and in bi-disperse sphere packing at two different velocities (200 and 500 µm/s), and their motion was simulated by the LPT/FB method. Thus, there were 35 simulations performed for this part of the investigation. To vary the NP concentration, both the particle size and the number of particles were adjusted as seen in Table 2. Results from these simulation runs were used to build the model showing the dependence of the aggregation rate on NP concentration. Aggregation rates obtained from two different concentrations of NPs (5.4 and 18.8 mg/L of CeO_2_ in 0.2 M KCl at 93 µm/s) aggregating in the tri-disperse packing were computed to verify the model predictions.

**Table 2:** The particle concentration in this work was changed by varying the number of particles and their respective sizes.

NP concentration (mg/L)	Number of particles	Single particle radius (nm)	Schmidt number
3.6	7809	75	349,447
5.3	7809	85	396,273
7.4	7809	95	443,100
10.0	10,544	95	443,100
14.0	14,720	95	443,100
18.8	14,720	105	489,926
27.4	21,428	105	489,926

To investigate if particles with different force field follow the same aggregation model as CeO_2_ NPs, additional LPT/FB runs were conducted for particles having Hamaker constant *A* = 5.57 × 10^−19^ J, ten times larger than that of CeO_2_. Four runs including10544 particles sized 95 nm and 7809 nanoparticles sized 105 nm moving through the mono-disperse sphere packing at pore velocities 200 and 500 µm/s were carried out to find the dependence of the aggregation rate on *Sc* and *Re*. Moreover, 7809 particles with radius of 75 nm, 7809 particles sized 85 nm, 14,725 particles sized 105 nm, and 21,428 particles sized 105 nm traveling through the same packing at 500 µm/s were simulated to investigate impacts of the particle concentration.

**Figure 2:**
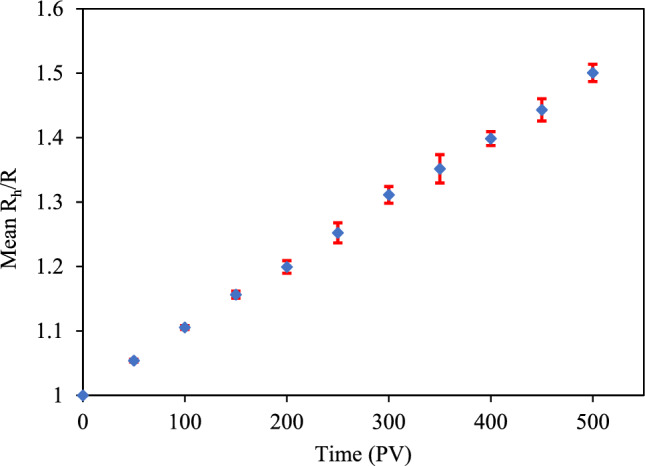
Mean value of the ratio of the hydrodynamic radius over the radius of the primary particle and the standard deviation of this ratio for six simulations simulating the aggregation of CeO_2_ nanoparticles in 0.2 M KCl. The nanoparticles flow and aggregate through mono-disperse spheres with pore velocity at 500 µm/s. The six simulations were performed with six different initial seeds for random number generation and with different initial positions of the particles. The maximum error is less than 3%.

While conducting multiple simulations for one case is required in probability-based models such as Monte Carlo, in our study, each case was simulated once. Because the model is not stochastic, and the use of probabilities is eliminated, performing multiple simulations for each case is unnecessary. To show the convergence of the model, six simulations of 10,544 CeO_2_ NPs (sized 95 nm) in 0.2 M KCl aqueous solution moving through a mono-disperse sphere packing at the pore velocity of 500 µm/s were carried out with different random numbers and different initial positions of particles. It is shown in Fig. 2 that the results from different simulations are not much different.

## Results

### Dependence of aggregation rate on time

**Figure 3:**
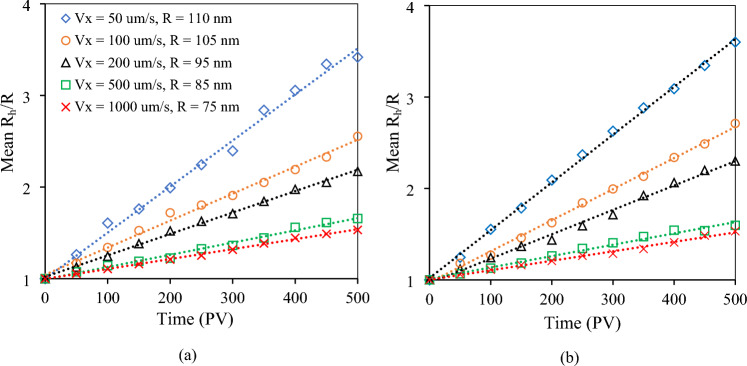
The relationship between the ratio of the mean hydrodynamic radius of NPs to the primary particle radius and time at different pore velocities and particle sizes when CeO_2_ NPs with a concentration of 10 mg/L flowed through (**a**) the mono-disperse sphere packing and (**b**) the bi-disperse sphere packing.

**Figure 4:**
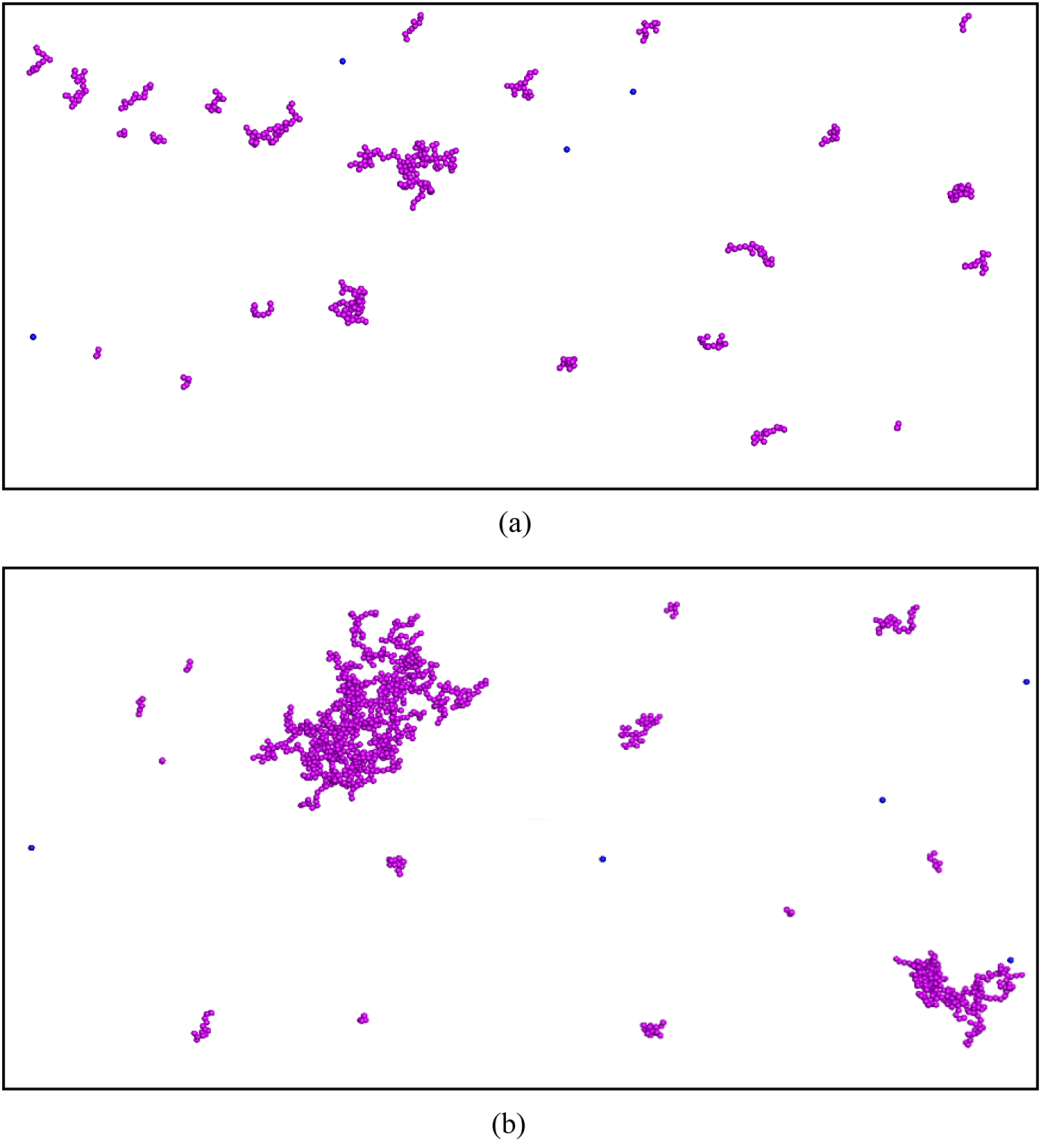
Typical clusters formed at (**a**) mean $\frac{{R}_{h}}{R}=3$ and (**b**) mean $\frac{{R}_{h}}{R}=5$ when 10,544 CeO_2_ NPs with the initial radius of 105 nm moved in the mono-disperse sphere packing at the velocity of 100 µm/s.

Figure 3 shows the ratio of the mean hydrodynamic radius of the particles over the primary particle radius (${R}_{h}/R$) for CeO_2_ NPs of different sizes and different fluid velocities in 0.2 M KCl solution as a function of time. Time is reported in terms of pore volumes, which illustrates how much fluid has been displaced through the porous medium. It was calculated by dividing the simulation time by the time that it takes for fluid to move through one simulation domain. Figure 3a is an illustration of the results from the experiments simulating the aggregation of NPs with the same particle concentration of 10 mg/L and different particle radii including 110, 105, 95, 85, and 75 nm when they moved in the mono-disperse sphere packing at different velocities 50, 100, 200, 500, and 1000 µm/s, respectively. The same runs in the bi-disperse sphere packing are displayed in Fig. 3b. It is clearly shown that when the time was less than 500 pore volumes the aggregation rate is linearly dependent on time, as below:

12 $$\frac{{R}_{h}}{R}=S\cdot t+1$$

where *S* is the slope of the line. In other words, the mean size of particles increased linearly with time as aggregation occurred. Figure 4a,b display typical images of clusters that were formed at the period when the mean ${R}_{h}/R$ was equal to 3 and 5, respectively. These results are for the case of 10,544 NPs with primary particle radius of *R* = 105 nm flowing through the mono-disperse sphere packing at a pore velocity in the flow direction, V_x_, of 100 µm/s. Different runs with different pore velocities and/or sizes of primary particles had different values of *S*, which showed that *S* depended on the Reynolds and Schmidt numbers at a constant NP concentration.

### Dependence of aggregation rate on Reynolds and Schmidt numbers

**Figure 5:**
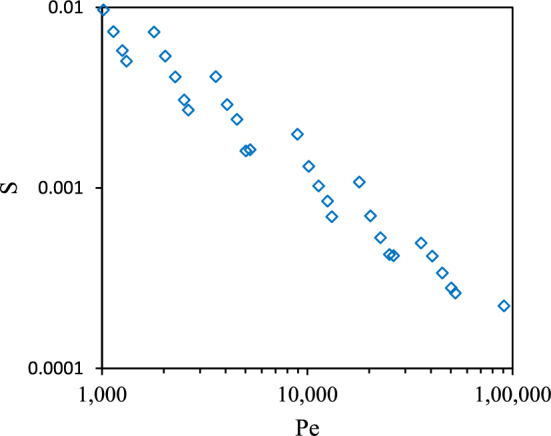
Dependence of the slope of the aggregation rate (S) on Peclet number for the case of CeO_2_ NPs moving through the monodisperse sphere packing.

**Figure 6:**
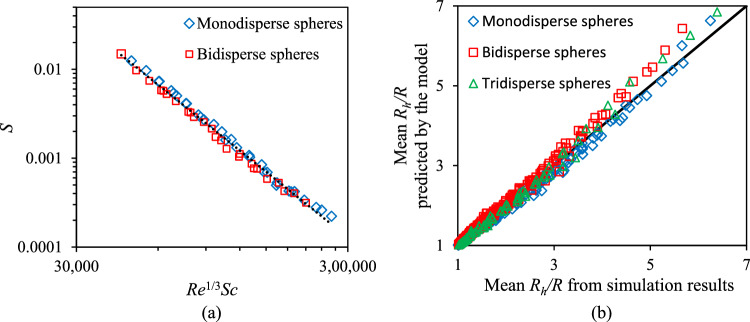
(**a**) Relationship between the slope of the aggregation rate (S) and Re^1/3^Sc when CeO_2_ NPs move through mono-disperse and bi-disperse sphere packings. Each point represents the S value of one simulation. (**b**) The comparison chart between aggregation rates obtained by simulations and those computed by Eq. (14). The black line in the chart is the unity line and each point is the aggregation rate at certain values of Re, Sc numbers, and time ranging from 50 to 500 PV with the increment of 50 PV.

Sixty simulations of 10 mg/L NPs (in 0.2 M KCl) with different particle radii (from 75 to 110 nm) moving in mono-disperse and bi-disperse sphere packings at different velocities ranging from 50 to 2000 µm/s were analyzed to examine how the *Re* and *Sc* numbers affected the value of the slope *S* appearing in Eq. (12) in particular, and the aggregation rate in general. The *Re* was calculated based on the hydraulic diameter of the porous medium (as the characteristic length scale) and the pore velocity in the flow direction, V_x_. The *Re* of fluid flows through the mono-disperse packing with a hydraulic diameter of 51.3 µm varied from 0.0026 to 0.1, while the *Re* for the bi-disperse packing having a hydraulic diameter of 39.7 µm was in the range of 0.002–0.079. The *Sc* corresponding to particles with radii from 75 to 110 nm fell in the range of 349,447 to 513,339. First, the effect of the Peclet number (*Pe* = *Re* × *Sc*) on the rate of change of the mean hydraulic radius, *S*, was investigated. As seen in Fig. 5, the slope of the aggregation rate and the Peclet number did not appear to be correlated. Thus, the effects of *Re* and *Sc* numbers were examined separately and it was found that the slope of the aggregation rate was correlated to *Re*^1/3^*Sc* for both mono-disperse and bi-disperse sphere packings, as seen in Fig. 6a.

The relationship between* S* and *Re*^1/3^*Sc* was found to be

13 $$S=6.0\times {10}^{9} {\left({Re}^{1/3}Sc\right)}^{-2.5}.$$

Therefore, when 10 mg/L CeO_2_ NPs traveled through mono-disperse and bi-disperse sphere packings, the mean hydrodynamic radius of particles divided by the primary particle radius could be expressed as follows:

14 $$\frac{{R}_{h}}{R}=6.0\times {10}^{9} {\left({Re}^{1/3}Sc\right)}^{-2.5}t+1.$$

The x-axis in Fig. 6b shows growth of the mean hydrodynamic radius estimated by simulation, while the y-axis represents the same parameter calculated by using Eq. (14). The black line in Fig. 6b is the identity line where the x-coordinate is equal to the y-coordinate. Each point on this chart represents the value of the aggregation rate of NPs for a specific combination of *Re, Sc,* and time. The aggregation rates shown in this chart were computed at different times from 50 to 500 PV with an increment of 50 PV. The proximity of a point in Fig. 6b to the identity line indicates the error between the aggregation rate predicted by Eq. (14) and the one estimated through simulation. The closer the point in Fig. 6b to the identity line, the more accurate the model. The maximum relative error and the average relative error for all points displayed in the chart were 13.6% and 2.7% respectively, which indicates that the proposed model in Eq. (14) gives a good prediction about the aggregation rate of 10 mg/L CeO_2_ NP based on *Re, Sc,* and time.

Equation (14) shows that the aggregation rate decreased when the Reynolds number increased. The Reynolds number represented the convection effects on aggregation due to fluid flow, and when it increased, the residence time of particles in the simulation domain was shorter. Thus, particles did not have enough time to interact or collide with each other and the aggregation rate was low. In addition, the aggregation rate also depended on the Schmidt number. A lower Schmidt number (corresponding to higher molecular diffusion of NPs) accelerated the collision efficiency, thereby improving the aggregation kinetics. However, the exponent of the *Sc* was three times as large as that of the *Re*, which showed that in diffusion-limited aggregation, the molecular diffusion had a much stronger effect on the aggregation kinetics than the convection effects.

### Dependence of aggregation rate on the particle concentration

**Figure 7:**
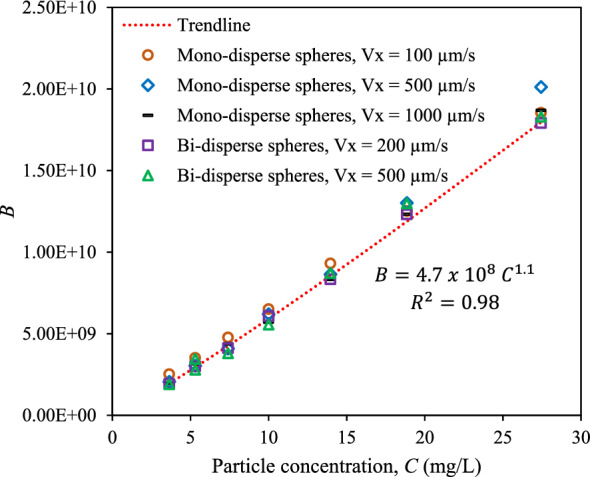
The relationship between the coefficient B and the particle concentration, C, when CeO_2_ NPs traveled through the mono-disperse sphere packing at velocities of 100, 500, and 1000 µm/s and through bi-disperse sphere packing at velocities of 200 and 500 µm/*s.*

Section “Dependence of aggregation rate on Reynolds and Schmidt numbers” shows the prediction model for the aggregation of 10 mg/L CeO_2_ NPs in 0.2 M KCl in sphere packings. When the concentration of the particles varies, the predictive equation should be expressed in a more general form as

15 $$\frac{{R}_{h}}{R}=B {\left({Re}^{1/3}Sc\right)}^{-2.5}t+1$$

where $B$ is a coefficient dependent on the concentration of NPs. In Eq. (14), it was *B* = $6.0\times {10}^{9}$, when the NP concentration was 10 mg/L. In this section, we aim to obtain the relationship between *B* and NP concentration by analyzing the results from numerical experiments with different concentrations at different velocities. It is seen in Fig. 7 that the values of *B* at different velocities and particle sizes were almost similar to each other. While the values of *B* were independent of *Re* and *Sc,* they correlated strongly to NP concentration, *C*. At very low NP concentration, *B* depended linearly on *C*, which is a result that agrees with the experimental results of Szilagyi et al.^44^ that examined the aggregation of nano-sized amidine latex particles suspended in ionic liquid and water mixture. It was found that when the particle concentration was less than 5 mg/L, the hydrodynamic radius change with time was a straight line and the gradient of the line was linearly correlated to the particle concentration. In our study, there was no linear correlation between the values of *B* and NP concentration for concentrations higher than 10 mg/L. We employed a power equation to fit all the data points in Fig. 7, so that the rate of CeO_2_ diffusion-limited aggregation in sphere packings can be modeled as

16 $$B=4.7\times {10}^{8} {C}^{1.1}$$

Therefore, the aggregate size can be predicted based on time, NP concentration, *Re*, and *Sc* by incorporating Eq. (16) in Eq. (15) as follows (when *C* is in mg/L):

17 $$\frac{{R}_{h}}{R}=4.7 \times {10}^{8} {C}^{1.1} {\left({Re}^{1/3}Sc\right)}^{-2.5}t+1$$

**Figure 8:**
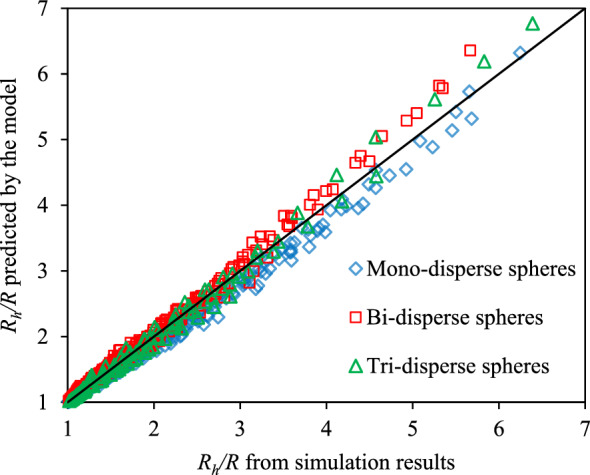
The comparison chart between aggregation rates obtained by simulations and those computed by Eq. (17). The black line in the chart is the unity line and each point is the aggregation rate at certain values of Re, Sc numbers, and time ranging from 50 to 500 PV with the increment of 50 PV.

Figure 8 is a chart comparing the aggregation rates predicted by Eq. (17) and those obtained at times ranging from 50 to 500 PV with an increment of 50 PV from all the simulations done for all three sphere packings as described in the scope of work. It is observed that the maximum relative error was 16.4% while the average relative error was 3.1%. Thus, Eq. (17) is a good model to predict the mean hydrodynamic radius of CeO_2_ NPs in the diffusion-controlled aggregation when they travel through sphere packings.

### Aggregation rate of particles with a different force field

**Figure 9:**
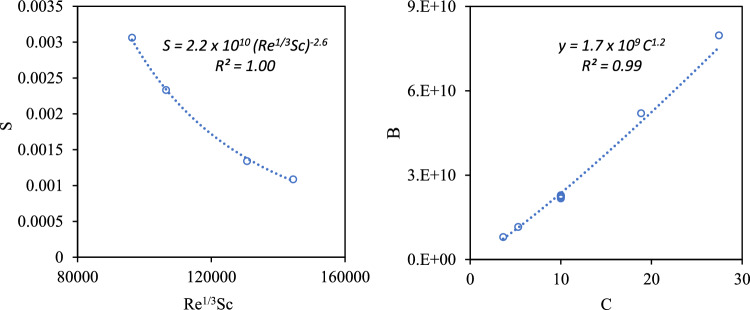
The relationship between (**a**) the slope of the aggregation rate, *S*, and *Re*^*1/3*^*Sc* when NPs (A = 5.57 × 10^−19^ J) sized from 95 to 105 nm, moved through mono-disperse packings at velocities of 200 and 500 µm, (**b**) the coefficient *B* and the particle concentration, *C*, when NPs (A = 5.57 × 10^−19^ J) with different concentrations ranging from 3.6 to 27.4 mg/L traveled through the mono-disperse sphere packing at the pore velocity of 500 µm.

Additional LPT/FB runs to compute the aggregation rates of particles other than CeO_2_ moving through the mono-disperse sphere packing were conducted. These particles were assumed to have Hamaker constant *A* = 5.57 × 10^−19^ J, which was an order of magnitude larger than the Hamaker constant of CeO_2_. From the dependence of *S* on *Re*^*1/3*^*Sc* and *B* on* C* as shown in Fig. 9a,b, the aggregation rate could be written as

18 $$\frac{{R}_{h}}{R}=1.7 \times {10}^{9} {C}^{1.2} {\left({Re}^{1/3}Sc\right)}^{-2.6}t+1.$$

It is found that the exponent of *Sc* is still three times larger than that of *Re* regardless of NP types, thus the impact of the particle size was more important than that of the flow field. Moreover, the exponents of NP concentration, *C*, (equal to 1.2) and the dimensionless number *Re*^*1/3*^*Sc* (equal to − 2.6), were very close to those of CeO_2_ (1.1 and − 2.5), even though the force field was far different. On the contrary, the force field strongly affected the scale factor, which was equal to $1.7\times {10}^{9}$ and $4.7 \times {10}^{8}$ when *A* = 5.57 × 10^−19^ J and *A* = 5.57 × 10^−20^ J, respectively.

## Conclusions

This work utilized the lattice Boltzmann method in conjunction with a Lagrangian particle tracking algorithm modified with the force balance approach to account for the interactions among particles to examine the effects of time, *Re*, *Sc*, and NP concentration on the mean aggregate size in the diffusion-limited regime when NPs moved through the pore space in randomly packed spheres. The aggregation rate can be expressed as a function of time in a generic form as follows:

19 $$\frac{{R}_{h}}{R}=\alpha {C}^{\beta }{\left({Re}^{1/3}Sc\right)}^{\gamma }t+1$$

where $$\alpha$$ is the scaling factor, $$\beta$$ and $$\gamma$$ are exponents for the particle concentration, *C,* and the dimensionless number ($${Re}^{1/3}Sc)$$. These parameters are expected to change when the interaction forces among particles vary. For the case of CeO_2_ NPs, when the concentration of NPs is less than 27.4 mg/L, Eq. (19) with $$\alpha =4.7\times {10}^{8}$$, $$\beta =1.1$$, and $$\gamma =- 2.5$$ could predict the aggregate sizes within acceptable accuracy—the average relative error was 3.1%. At very dilute NP concentrations (< 10 mg/L CeO_2_), $$\beta$$ is equal to 1. These findings are in agreement with experiments from another laboratory where a linear correlation between aggregate size and NP concentration was reported^44^. The rate of diffusion-limited aggregation is strongly affected by the particle size (i.e., Schmidt number) and this effect was three times as strong as the convection effect (i.e., Reynolds number). Thus, it is proved herein that the aggregation process of particles with known physicochemical properties (such as Hamaker constant, surface potential, and size) could be estimated and controlled by the size of primary particles, the particle concentration, and the flow field in decreasing order of influence. Thus, future work focusing on how to find parameters $$\alpha$$, $$\beta$$ and $$\gamma$$ based on the particle force fields is essential, since it helps to quickly predict the aggregation rate of NPs in porous media for different types of NPs in different solutions. In addition, the particle–wall interaction is not considered in this study. Thus, it is suitable when the particle–wall interaction is either insignificant or very small compared to the particle–particle interaction. Such interactions can be incorporated in future work.

## Data Availability

The datasets used and/or analyzed during the current study are included herein. Raw data can be available from the corresponding author upon reasonable request.
